# Robotic-assisted lymphatic surgery for lymphedema: Bibliometric mapping of an emerging reconstructive field

**DOI:** 10.1016/j.jpra.2026.08.025

**Published:** 2026-08-21

**Authors:** Adriano Fabi, Séverin R Wendelspiess, André S Alves, Felix J Klimitz, Shan Shan Qiu, Siba Haykal, Elisabeth A Kappos

**Affiliations:** a Department of Plastic, Reconstructive, Aesthetic and Hand Surgery, University Hospital Basel, Basel, Switzerland; b Faculty of Medicine, University of Basel, Basel, Switzerland; c Department of Surgery, Cantonal Hospital of Lucerne, Lucerne, Switzerland; d Department of Plastic, Reconstructive and Aesthetic Surgery, Geneva University Hospitals, Geneva University, Geneva, Switzerland; e Division of Plastic and Reconstructive Surgery, Department of Surgery, Yale School of Medicine, Yale New Haven Hospital, CT, USA; f Department of Plastic, Reconstructive and Hand Surgery, Maastricht University Medical Center, Maastricht, the Netherlands

**Keywords:** Robotic surgery, Supermicrosurgery, Lymphatic reconstruction, Lymphovenous anastomosis, Vascularized lymph node transfer, Lymphedema, Microsurgical innovation

## Abstract

**Background:**

Lymphatic reconstruction, including lymphovenous anastomosis (LVA) and vascularized lymph node transfer (VLNT), is an established surgical treatment for chronic lymphedema. However, these procedures rely on supermicrosurgical manipulation of submillimeter vessels, rendering outcomes highly dependent on surgeon dexterity and technical precision. Robotic platforms have recently been introduced to enhance stability and tremor filtration. However, the evidence and true patient benefit of the existing literature remain subject of ongoing debate.

**Methods:**

A bibliometric analysis of the Web of Science Core Collection was conducted to identify publications on robotic-assisted lymphatic reconstruction from database inception to February 2026. Eligible records were manually screened. Publication trends, levels of evidence, citation metrics, geographic distribution, and collaborative networks were analyzed using VOSviewer.

**Results:**

Thirty-five studies met the inclusion criteria. Publication activity became more consistent from 2023 onward, accounting for 71.4% of all articles. The evidence base was dominated by low-quality studies, with level V research comprising 68.6% of publications. Notably, research output was concentrated within a limited number of high-volume centers, primarily located in the United States and Switzerland, with little international collaboration.

**Conclusions:**

Robotic-assisted lymphatic surgery for lymphedema is a rapidly emerging field characterized by increasing publication activity, but limited quality of evidence. Current studies support technical feasibility in selected settings, but remain insufficient to establish clinical superiority or cost-effectiveness compared with manual surgery. The field is currently driven by technological innovation rather than high-level clinical validation. Prospective comparative studies are required to define the role of robotic platforms in physiological lymphatic reconstruction.

## Introduction

Lymphedema is a chronic, progressive condition resulting from impaired lymphatic drainage, leading to swelling, pain, functional limitations, and reduced quality of life.^1^, ^2^, ^3^ While complex decongestive therapy (CDT), including compression, manual lymph drainage, exercise, and skin care, remains the first-line approach, it does not address the underlying pathophysiology of lymphatic insufficiency.^4^

Over the last decade, physiologic surgical approaches, such as lymphovenous anastomosis (LVA) or vascularized lymph node transfer (VLNT), have become established components of surgical lymphedema treatment and have shown significant reductions in limb volume, compression dependence, alongside improved patient-reported quality of life.^5^, ^6^, ^7^, ^8^

Supermicrosurgery enables anastomoses of lymphatic and venous structures in the submillimeter range, but such precision pushes the limits of human dexterity and is highly sensitive to tremor, fatigue and ergonomic constraints.^9^ To overcome these limitations, robotic platforms have been developed to provide tremor filtration and enhanced access into deep or confined anatomical regions.^10^^,^^11^ Early clinical experiences demonstrate that robot assisted supermicrosurgical procedures are technically feasible and safe.^12^^,^^13^ However, widespread adoption remains limited by very high acquisition costs, lack of haptic feedback, and non-inferiority of manual lymphatic microsurgery.^9^^,^^14^

Despite growing interest in robotic assistance for lymphatic surgery, only a small number of centers currently employ robotic platforms, and the quality of the supporting evidence base has not been systematically characterized. Consequently, the goal of this study was to map the current literature, publication trends, collaborative patterns and quality of evidence in robotic-assisted lymphatic reconstruction, thereby providing an objective assessment of the current state and future direction of this emerging field.

## Methods

This study was designed as a bibliometric mapping study using a predefined search strategy and eligibility framework. Because the objective was to characterize the current research landscape rather than to systematically analyze treatment effectiveness, this study was not registered in a review database.

### Search strategy

The literature search was conducted using Clarivate’s Web of Science Core Collection (WOSCC) from database inception to February 1, 2026. The Web of Science Core Collection was selected because it provides standardized bibliographic and cited-reference metadata that can be exported directly into VOSviewer with consistent field structure. The search aimed to identify publications addressing robotic-assisted lymphatic surgery in the context of lymphedema. Articles were eligible if they focused primarily on robotic-assisted lymphatic surgery, or if the use of robotic systems was described as a clearly defined subsection within the article. Publications focusing exclusively on robotic surgery for oncologic lymph node dissection or unrelated surgical fields were excluded. Only articles with English-language abstract and keywords were included to ensure consistency in keyword, citation, and network analyses. The exclusion terms were added after exploratory testing of the broader search strategy. The search without the NOT block retrieved a largely similar set of relevant records, together with a small number of clearly unrelated publications from other robotic surgical fields. The NOT terms were therefore used to reduce predictable search noise and improve screening efficiency. The following final topic search (TS) strategy was applied:

TS=((robot* OR "robotic-assisted" OR "robot-assisted" OR "robotic surgery" OR musa OR symani) AND (lymphedema OR lymphoedema OR "lymphovenous anastomosis" OR "lymphaticovenular anastomosis" OR LVA OR "vascularized lymph node transfer" OR VLNT OR "lymph node transfer" OR “lymphatic reconstruction” OR VLVT OR LNVA)) NOT TS=(prostate OR urolog* OR penis OR colorectal OR thoracic OR lung OR gastric OR esophagectomy OR hysterectomy OR gynecolog* OR “general surgery” OR hepatobiliary OR pancreas OR pancreatic OR liver OR thyroidectomy OR cystectomy OR sclerosis OR simvastatin OR polyurethane OR rehabilitation OR lymphadenectomy OR “endometrial cancer” OR “mobilization device” OR trachelectomy OR Actuators)

### Study selection and classification

All retrieved records were screened for relevance based on title/abstract and full-text review. Publications were included if they addressed robotic assisted lymphatic surgery for lymphedema, either as a principal focus of the article or as a distinct subsection. Title/abstract and full-text eligibility assessments were performed by one reviewer. The use of a single reviewer was considered pragmatic given the narrow eligibility framework, the limited number of retrieved records, and the high specificity of the search strategy. To provide a descriptive estimate of evidence maturity, each included publication was additionally assigned an Oxford’s level of evidence (LOE) grade.^15^^,^^16^ Study quality classification, including assignment of Oxford levels of evidence, was performed by a second reviewer. Any uncertainties regarding eligibility or classification were resolved by discussion and consensus. Due to the heterogeneous article types, LOE was used as a pragmatic descriptive tool rather than as a formal comparative quality assessment. Because eligibility screening was not performed independently by two reviewers, no inter-reviewer agreement statistic was calculated.

### Data collection and analysis

For each publication, the following bibliometric metadata were extracted from WOSCC: title, authors, abstract, keywords, year of publication, journal, and cited references. All records were exported in plain text format for further analysis. Before bibliometric analysis, all author names were systematically reviewed for spelling variants, transliteration differences, diacritics, initials, and surname formatting. A VOSviewer thesaurus file was then applied to merge duplicate variants under standardized author names. Bibliometric and network analyses were performed using VOSviewer (version 1.6.20) to visualize publication trends, country-level contributions, journal distribution, and bibliographic relationships using full counting. Given the limited number of included publications, the minimum inclusion threshold was set at one article for all visual analyses. The final search was run in the Web of Science Core Collection on February 1, 2026, and the complete records and cited references were exported on the same date. Link strengths were normalized using the association-strength method.

## Results

### Global publication trends

A total of 35 publications addressing the use of robotic surgery in lymphatic reconstruction were identified in the WOSCC. No records were further excluded after screening following application of the predefined search strategy. Notably, publication activity was limited before 2023, after which the field entered a period of sustained output (Table 1). More than one quarter of the entire body of literature (25.7%) appeared in 2025 alone, whereas annual publication output before 2023 ranged from only one to five articles. Collectively, the included publications received 434 citations, corresponding to a mean of 12.4 citations per article.

**Table 1:** Bibliometric overview of all included articles.

**Characteristic**	**n (%)**
*Article Type*	
Literature Review	15 (42.9%)
Case Series	5 (14.3%)
Case Report	5 (14.3%)
Expert Opinion	4 (11.4%)
Systematic Review	3 (8.6%)
Randomized Study	2 (5.7%)
Retrospective Cohort Study	1 (2.9%)

*Level of Evidence*	
I	0 (0%)
II	3 (8.6%)
III	3 (8.6%)
IV	5 (14.3%)
V	24 (68.6%)

*Publication Year*	
2025	9 (25.7%)
2024	8 (22.9%)
2023	8 (22.9%)
2022	1 (2.9%)
2021	5 (14.3%)
2020	3 (8.6%)
2017	1 (2.9%)

*Journal*	
Plastic and Reconstructive Surgery – Global Open	4 (11.4%)
Journal of Robotic Surgery	3 (8.6%)
Plastic and Aesthetic Research	3 (8.6%)
Microsurgery	2 (5.7%)
Current Oncology	2 (5.7%)
Gland Surgery	2 (5.7%)
Seminars in Plastic Surgery	2 (5.7%)
Journal of Craniofacial Surgery	2 (5.7%)
Clinics in Plastic Surgery	2 (5.7%)
Journal of Reconstructive Microsurgery	1 (2.9%)
Annals of Plastic Surgery	1 (2.9%)
Journal of Plastic Reconstructive and Aesthetic Surgery	1 (2.9%)
British Journal of Surgery	1 (2.9%)
Journal of Clinical Medicine	1 (2.9%)
BMJ Case Reports	1 (2.9%)
Handchirurgie Mikrochirurgie Plastische Chirurgie	1 (2.9%)
European Journal of Plastic Surgery	1 (2.9%)
Plastic and Reconstructive Surgery	1 (2.9%)
Breast Care	1 (2.9%)
Asian Journal of Surgery	1 (2.9%)
Lancet Oncology	1 (2.9%)
Nature Communications	1 (2.9%)

### Predominance of low-level clinical evidence

Although reviews and expert-opinion articles constituted a substantial proportion of the literature, the primary clinical evidence was also methodologically limited, consisting mainly of small case reports, case series, feasibility studies, and non-comparative observational designs (Table 1). The majority of studies were classified as level V evidence (n = 24, 68.6%). Consistent with this distribution, literature reviews constituted the most frequent article type (42.9%), followed by case series and case reports, which together accounted for 28.6% of publications. Only six studies (17.1%) met criteria for higher-level evidence, including systematic reviews, randomized studies, or retrospective cohort analyses. No level I studies were identified.

### Concentration of research output among high-volume centers

**Fig. 1:**
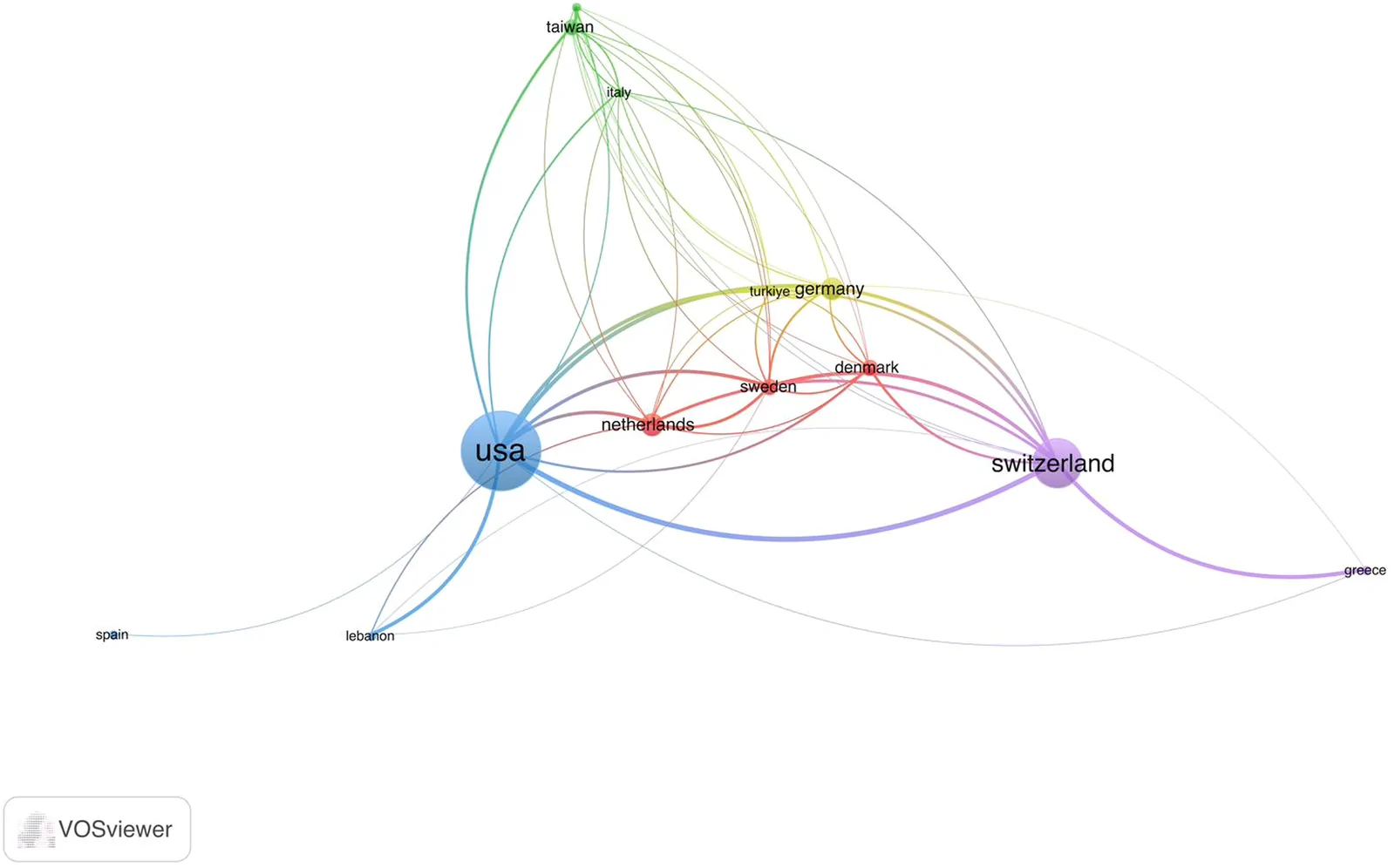
Countries performing research on robotic systems in lymphedema. Node size represents country frequency, and links indicate co-occurrence within individual publications, highlighting international collaborations.

The 35 included publications involved contributions from 151 authors across 54 institutions in 15 different countries. However, research output was highly concentrated among a limited number of centers. The United States emerged as the leading contributor in the number of publications, followed closely by Switzerland, with bibliometric visualization confirming close bilateral collaboration between these two countries, which formed the dominant research network, against a background of otherwise limited international collaboration (Fig. 1). Other countries, including the Netherlands, Germany, and Taiwan, participated in the collaborative publications, but did not emerge as independent research hubs to date. At the author level, Nicole Lindenblatt (n = 5, 14.3%) and collaborators from the University Hospital Zurich emerged as the most productive researchers in terms of quantitative output. Other leading figures included Shan Qiu and Jesse C Selber, each contributing three (8.6%) articles.

### Journal distribution

The literature on robotic surgery for lymphatic reconstruction was dispersed across 22 journals (Table 1). *Plastic and Reconstructive Surgery – Global Open* published the largest number of articles (n = 4, 11.4%), followed closely by the *Journal of Robotic Surgery* and *Plastic and Aesthetic Research*, with 3 (8.6%) articles each. Remaining publications were distributed across a wider range of surgical, oncological, and specialty journals. Temporal analysis of journal distribution found that more recent studies increasingly appeared in established plastic and reconstructive surgery journals.

### Most influential publications

**Fig. 2:**
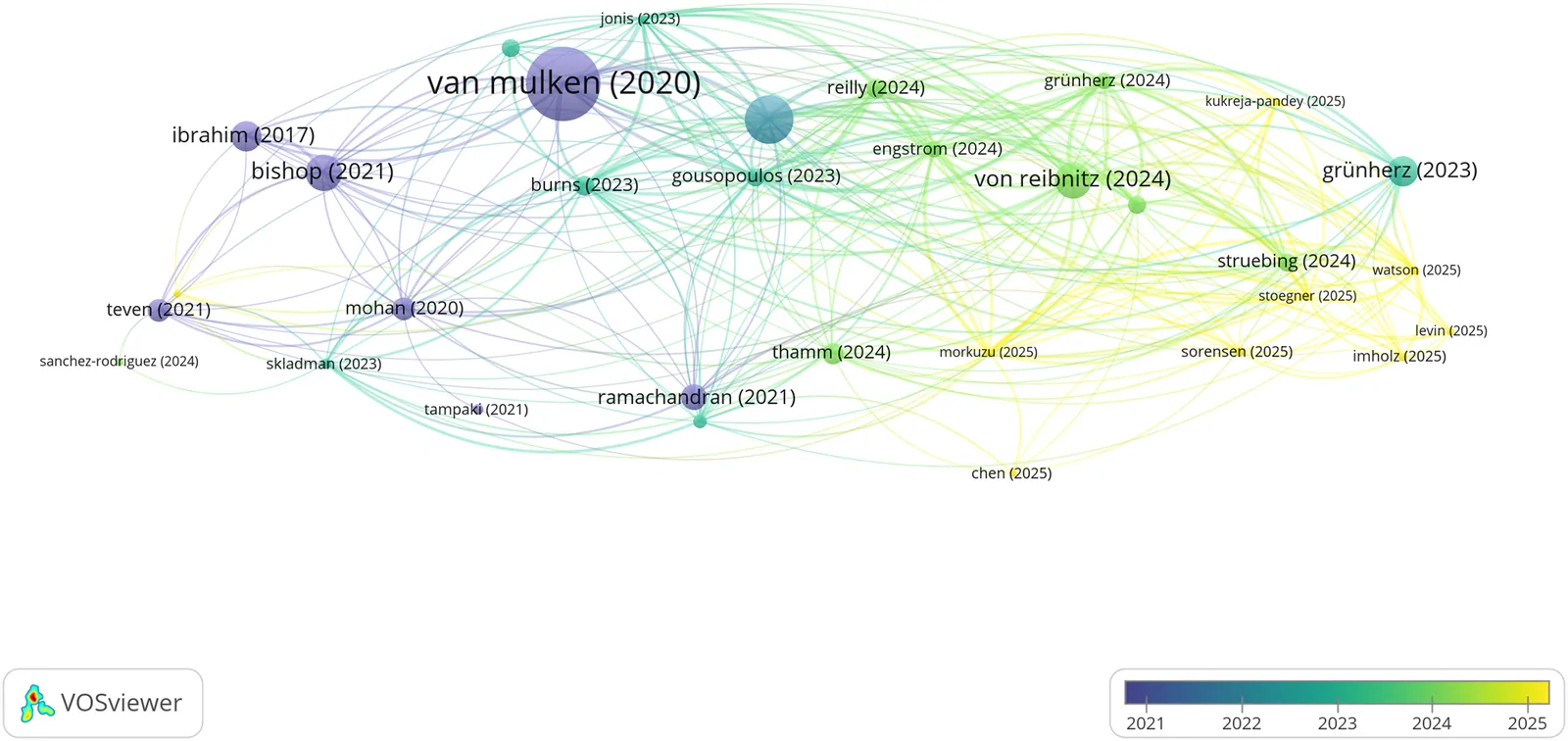
Bibliographic coupling of the most influential articles. Each node represents an individual publication, with node size proportional to the number of citations within the dataset. Links between nodes indicate shared references, and the strength of connections reflects the degree of bibliographic coupling. The overlay visualization displays the average publication year, with earlier studies shown in darker colors and more recent publications shown in lighter colors.

Bibliographic coupling identified a small number of landmark papers that have disproportionately shaped the field of robotic surgery (Fig. 2). The most cited study was the 2020 randomized controlled trial (RCT) by van Mulken et al., titled “*First-in-human robotic supermicrosurgery using a dedicated microsurgical robot for treating breast cancer-related lymphedema: a randomized pilot trial*” published in Nature Communications (Table 2).^17^ By comparing robot-assisted versus conventional manual lymphovenous anastomosis (LVA), demonstrating technical feasibility and safety, as well as comparable short-term improvements in arm volume and quality of life, this study accumulated 128 citations to date. The second most cited publication, also authored by van Mulken et al., reported one-year outcomes from the same patient cohort and demonstrated sustained quality-of-life improvements at 12 months, without evidence of clinical superiority of robotic over manual LVA.^18^ The third most influential study was the single-center case series by von Reibnitz et al.’s*“100 anastomoses: a two-year single-center experience with robotic-assisted micro- and supermicrosurgery for lymphatic reconstruction”* published in the Journal of Robotic Surgery.^19^ This report, the largest series to date, further supported feasibility, but similarly did not demonstrate clinical advantages associated with robotic-assisted lymphatic reconstruction, consequently receiving 31 citations. Notably, bibliographic coupling revealed a highly centralized intellectual structure (Fig. 2). A dominant core cluster was centered around the pioneering randomized controlled trial by van Mulken et al., which served as the principal article linking the majority of subsequent publications.^17^ Later studies demonstrated strong coupling to this foundational work rather than forming independent thematic clusters, indicating centralization. No distinct thematic clusters addressing cost-effectiveness, learning curves, or long-term patient-reported outcomes were identified.

**Table 2:** All included articles ranked by total citation count.

**Title**	**Author**	**Journal**	**Year**	**Citations**	**C/Y**	**LOE**
First-in-human robotic supermicrosurgery using a dedicated microsurgical robot for treating breast cancer-related lymphedema: a randomized pilot trial	van Mulken et al.	Nature Communications	2020	128	25.6	II
One-Year Outcomes of the First Human Trial on Robot-Assisted Lymphaticovenous Anastomosis for Breast Cancer-Related Lymphedema	van Mulken et al.	Plastic and Reconstructive Surgery	2022	53	17.7	II
100 anastomoses: a two-year single-center experience with robotic-assisted micro- and supermicrosurgery for lymphatic reconstruction	von Reibnitz et al.	Journal of Robotic Surgery	2024	31	31	IV
Minimally invasive robotic breast reconstruction surgery	Bishop et al.	Gland Surgery	2021	31	7.8	V
First-in-human Use of a Microsurgical Robotic System for Central Lymphatic Reconstruction	Grünherz et al.	Plastic and Reconstructive Surgery – Global Open	2023	22	11	V
New Frontiers in Robotic-Assisted Microsurgical Reconstruction	Ibrahim et al.	Clinics in Plastic Surgery	2017	22	2.8	V
Current operative management and therapeutic algorithm of lymphedema in the lower extremities	Ramachandran et al.	Asian Journal of Surgery	2021	15	3.8	V
Expanding the Horizon: Single-port Robotic Vascularized Omentum Lymphatic Transplant	Teven et al.	Plastic and Reconstructive Surgery – Global Open	2021	12	3	V
Recent Advances in Microsurgery: An Update in the Past 4 Years	Mohan et al.	Clinics in Plastic Surgery	2020	12	2.4	V
Advances in Modern Microsurgery	Thamm et al.	Journal of Clinical Medicine	2024	11	11	V
Use of the BHS robotic scope to perform lymphovenous anastomosis	Scaglioni et al.	Microsurgery	2021	11	2.8	V
Robotics in Microsurgery and Supermicrosurgery	Burns et al.	Seminars in Plastic Surgery	2023	10	5	V
Implementation of robot-assisted lymphaticovenous anastomoses in a microsurgical unit	Reilly et al.	European Journal of Plastic Surgery	2024	9	9	IV
Robot-assisted microsurgery: a single-center experience of 100 cases	Struebing et al.	Journal of Robotic Surgery	2024	8	8	IV
Robot-assisted lymphovenous anastomosis surgery for lymphocele in the groin	Lilja et al.	BMJ Case Reports	2024	8	8	V
The Full Continuum of Robotic Breast Surgery: Robotic-assisted Mastectomy, Robotic DIEP Flap, and Robotic Supermicrosurgery	Tanna et al.	Plastic and Reconstructive Surgery – Global Open	2023	8	4	V
Robotic-assisted microsurgery for lymphedema treatment	Gousopoulos et al.	Plastic and Aesthetic Research	2023	8	4	III
Surgical treatments of lymphedema-a literature review on robot-assisted lymphovenous anastomosis (LVA)	Engstrom et al.	Gland Surgery	2024	7	7	V
Robotic-Assisted Lymphatic Surgery	Grünherz et al.	Handchirurgie, Mikrochirurgie, Plastische Chirurgie	2024	7	7	V
Diagnosis and Treatment of Post-Prostatectomy Lymphedema: What's New?	Bianchi et al.	Current Oncology	2023	5	2.5	V
Evaluation of microsurgical free flap procedures utilizing the Symani Surgical System: A preliminary analysis	Sorensen et al.	Journal of Plastic, Reconstructive and Aesthetic Surgery	2025	3	3	IV
Vascularized omental tissue transfer for the treatment of lymphedema: a review	Skladman et al.	Plastic and Aesthetic Research	2023	3	1.5	V
Breast Reconstruction: Necessity for Further Standardization of the Current Surgical Techniques Attempting to Facilitate Scientific Evaluation and Select Tailored Individualized Procedures Optimizing Patient Satisfaction	Tampaki et al.	Breast Care	2021	3	0.8	V
Robotic-assisted lymphovenous anastomosis to treat periorbital lymphedema and systematic review of lymphatic reconstruction of face and neck lymphedema	Imholz et al.	Journal of Robotic Surgery	2025	2	2	III
The MUSA robot and its applicability in lymphatic surgery	Jonis et al.	Plastic and Aesthetic Research	2023	2	1	V
Robot-assisted supermicrosurgery for lymphoedema	Gourd	Lancet Oncology	2020	2	0.4	V
Robot-Assisted Lymph Node-to-Vein Anastomosis: Lessons from the First 22 Cases at a High-Volume Lymphatic Supermicrosurgery Center	Chen et al.	Current Oncology	2025	1	1	IV
Robotic-Assisted Lymphovenous Anastomosis: A Systematic Review of Surgical Outcomes	Morkuzu et al.	Microsurgery	2025	0	0	III
Single Port Robotic Vascularized Omental Lymph Node Transfer for Lymphedema: A Novel Comparison to Open Technique	Jeger et al.	Journal of Reconstructive Microsurgery	2025	0	0	II
Brain Drain for Brain Gain: Potential Applications of Robotic-assisted Lymphatic Microsurgery in the Management of Neurological Disorders	Watson et al.	Plastic and Reconstructive Surgery – Global Open	2025	0	0	V
Robotic-Assisted Lymphatic Supermicrosurgery for Breast Lymphedema-A Case Report and Literature Review	Kukreja-Pandey et al.	Annals of Plastic Surgery	2025	0	0	V
Robotic-Assisted Lymphedema Surgery: Bridging the Gap in Training and Expanding Complex Surgical Options	Levin et al.	Journal of Craniofacial Surgery	2025	0	0	V
Robotic-Assisted Microsurgery in Lymphatic Reconstruction	Stoegner et al.	Journal of Craniofacial Surgery	2025	0	0	V
Combined pedicled and free omentum flaps for chronic lower limb lymphoedema: novel approach with robotic surgery	Sanchez-Rodriguez et al.	British Journal of Surgery	2024	0	0	V
Applications for Robotic Surgery within Lymphedema, Pelvic Recon, and Breast	Yu et al.	Seminars in Plastic Surgery	2023	0	0	V

*C/Y* Citations per year; *LOE* Level of Evidence.

## Discussion

To the best of our knowledge, this study represents the first bibliometric mapping of the literature on robotic-assisted lymphatic surgery for lymphedema. Although publication activity has become more consistent in recent years, the literature remains dominated by non-comparative and secondary publications, concentrated within a limited number of expert groups, and focused primarily on feasibility rather than comparative effectiveness. These findings suggest that the field has not yet reached a stage of evidence-based clinical adoption.

A central finding of this study is the predominance of secondary publications and expert opinions, with more than two thirds of included publications classified as level V evidence. This distribution is consistent with the developmental stage of the field, particularly given that dedicated microsurgical robotic systems have only recently entered clinical use. Most publications consist of feasibility reports, technical notes and early clinical experience, whereas higher-level comparative studies remain scarce. This interpretation is further supported by the bibliographic structure of the field, in which a small number of early landmark publications continue to serve as the main reference points for much of the subsequent literature.

From a clinical perspective, the current literature should therefore be interpreted cautiously. Because most available studies are early-phase or observational, any reported advantages may reflect selection bias and early-adopter enthusiasm or learning curve effects. Another important consideration is that robotic-assisted lymphatic surgery is not a single uniform entity. Applications range from dedicated microsurgical robots used for peripheral supermicrosurgical anastomoses, robotic visualization systems, or robotic approaches to central lymphatic reconstruction, with the current literature mostly focusing on peripheral lymphovenous anastomosis.^11^

Geographically, research activity was concentrated in a limited number of high-resource centers, primarily in the United States and Switzerland. This pattern likely reflects the restricted availability of robotic platforms, the technical specialization required for robotic microsurgery, and the fact that early clinical implementation has occurred in only a small number of lymphedema centers, reflecting institutional and financial requirements associated with robotic platforms, including capital investment, maintenance, and specialized training, which are more commonly available in high-resource healthcare systems.^20^ The concentration of output within a small collaborative network further reinforces the early-phase nature of the field. Importantly, this pattern can be interpreted as reflecting unequal access and implementation capacity, rather than limited global interest in robotic-assisted lymphatic surgery.

With regard to clinical effectiveness, the currently available data do not demonstrate superiority of robotic-assisted LVA over conventional manual techniques.^17^^,^^18^ While robotic systems may offer technical benefits such as precision and tremor filtration, these theoretical advantages appear to come at the expense of substantially longer operative times and potentially higher procedural costs.^21^ It is critical to distinguish, however, between absence of evidence for superiority and definitive evidence of equivalence.^22^ Current data are simply insufficient to support widespread adoption on outcome-based grounds, but they are also insufficient to exclude potential benefit in selected indications. In many respects, robot-assisted lymphatic surgery appears to mirror the early trajectory observed in other robotic surgical disciplines, where initial growth was driven by concentrated expertise and institutional interest before higher-level comparative evidence became available.^23^

Three important questions remain unanswered by the current literature and remain central to the value assessment of robotic surgery. First, the learning curve for robotic supermicrosurgery is not defined; although robotic assistance is intended to reduce the manual dexterity required, mastering the platform itself may be demanding, and the number of cases needed to reach proficiency has not been established. Second, robotic assistance may potentially require longer total operative times due to set-up. Third, robust comparative cost analyses, accounting for capital investment, maintenance, disposables and operating-room time at the level of the patient, institution and health-care system, are largely lacking. Consequently, future research should prioritize well-designed multicenter prospective studies to improve generalizability and reduce single-center bias. Rather than pursuing generalized application, future work should aim to identify specific clinical scenarios, in which robotic assistance may provide benefit, such as central lymphatic reconstruction, and explore emerging translational domains, including neurolymphatic surgery.^24^, ^25^, ^26^

### Limitations

This is the first bibliometric analysis discussing the development of robotic supermicrosurgery in lymphedema over time. However, the analysis was restricted to the Web of Science Core Collection. Although this database includes the majority of scientific literature and provides standardized metadata suitable for VOSviewer analyses, other articles exclusively indexed in Scopus, PubMed/MEDLINE or Embas may not have been captured. Secondly, as only English-language publications were included to allow for keyword analysis, this may potentially underrepresent contributions from non-English-speaking countries. Furthermore, study screening was performed by a single reviewer, which may have introduced selection bias, despite pre-defined inclusion criteria. Additionally, the included records were methodologically heterogeneous and, accordingly, level-of-evidence grading should be interpreted as a descriptive estimate rather than a formal quality comparison across study designs. Bibliometric analyses are inherently influenced by publication timing and citation accrual, such that early landmark papers may appear disproportionately influential relative to more recent publications. Finally, the present study maps the structure of the literature rather than measuring clinical effectiveness directly. Therefore, bibliometric prominence must not be interpreted as proof of clinical value.

## Conclusion

Robotic-assisted lymphatic surgery is an emerging field with increasing publication activity but limited comparative primary evidence. Current studies support early clinical use in selected settings but remain insufficient to establish clinical superiority or cost-effectiveness compared with conventional manual lymphatic surgery. The next phase of development will require high-quality comparative studies to determine whether this innovation can achieve clinical relevance and wider accessibility.

## Data availability

The bibliometric data underlying this study were obtained from the Web of Science Core Collection using the search strategy described in the Methods. As Web of Science is a subscription-based database, access to the original records may require institutional access. The extracted dataset used for analysis is available from the corresponding author upon reasonable request.

## Funding

This study received no external funding.

## Ethics approval

Not applicable.

## Patient consent to participate

Not applicable.

## CRediT authorship contribution statement

**Adriano Fabi:** Conceptualization, Writing – review & editing, Data curation, Formal analysis, Writing – original draft, Project administration, Supervision. **Séverin R Wendelspiess:** Conceptualization, Writing – original draft. **André S Alves:** Conceptualization, Visualization. **Felix J Klimitz:** Conceptualization. **Shan Shan Qiu:** Conceptualization. **Siba Haykal:** Conceptualization. **Elisabeth A Kappos:** Conceptualization.

## Conflicts of interest

Siba Haykal is a consultant for Medical Microinstruments Inc (MMI). None of the other authors have any conflicts of interests to disclose.
